# Electrochemical Detection of Perfluorooctanoic Acid by a Zn–Terpyridine and Nafion‐Modified Glassy Carbon Electrode

**DOI:** 10.1002/cplu.70272

**Published:** 2026-10-20

**Authors:** Alyah Buzid, Kim J. Keohane, Sarah Cramp, Christopher S. Burke

**Affiliations:** ^1^ Department of Chemistry College of Science King Faisal University Al‐Ahsa Saudi Arabia; ^2^ School of Chemistry and Analytical & Biological Chemistry Research Facility (ABCRF) University College Cork Cork Ireland

**Keywords:** drinking water, electrochemical sensor, environmental monitoring, per‐ and polyfluoroalkyl substances, PFAS, PFOA, zinc terpyridine complex

## Abstract

Perfluorooctanoic acid (PFOA), a persistent organic pollutant, poses significant environmental and health risks due to its widespread occurrence in water sources and resistance to degradation. This study investigates zinc(II) terpyridine (Zn–tpy) complexes as molecular receptors for PFOA, demonstrating capture via carboxylate binding to Zn(II) with concomitant changes to nuclear magnetic resonance (NMR) and absorbance spectra. The capture capability and redox properties of the complexes were used to develop an electrochemical method for PFOA at a glassy carbon (GC) electrode modified with a thin discontinuous Nafion overlayer that assists with preconcentration at the sensor. Cyclic voltammetry (CV), electrochemical impedance spectroscopy (EIS), and square‐wave voltammetry (SWV) were used to characterize the modified electrode. The electrode gave a limit of detection (LOD) of 0.407 pM and a linear range of 0.5–10 pM, with short‐term intra‐ and interelectrode relative standard deviations (RSD) of 0.94% and 2.02%. Recoveries of 96%–98% were obtained for Cork tap water spiked with PFOA after 1:100 dilution in phosphate‐buffered saline (PBS). The preliminary data suggests Zn–tpy complexes in combination with selective coatings may be applicable for class‐selective electrochemical detection of perfluoroalkyl carboxylic acids, exemplified herein using PFOA.

## Graphical abstract

PFOA can substitute acetate ligands in zinc terpyridine complexes to elicit spectroscopic changes. This phenomenon in solution is exploited for electrochemical sensing using discontinuous Nafion overlayer as a preconcentrating membrane to provide detection limits of 0.407 pM and a linear range of 0.5–10 pM. The data suggests this approach to be useful for screening perfluoroalkylcarboxylic acids in drinking water.

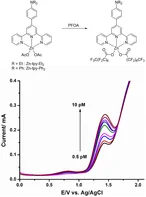

## 1 Introduction

Perfluorooctanoic acid (PFOA) is a synthetic chemical belonging to the group of per‐ and polyfluoroalkyl substances (PFAS). Since its introduction in the 1950s, PFOA has been extensively used in various industrial applications, including the production of fluoropolymers, which are essential in manufacturing nonstick cookware, water‐repellent fabrics, and firefighting foams [1, 2]. The widespread use of PFOA in these industries has led to its pervasive presence in the environment. PFOA is highly persistent, resisting natural degradation processes, which results in its accumulation in water, soil, and living organisms [3]. The environmental persistence and bioaccumulation of PFOA have raised significant concerns. PFOA was detected in the blood of humans and wildlife globally, indicating its widespread distribution [4]. The presence of high levels of PFOA in drinking water sources is a significant cause for concern as it poses potential health risks to humans, including developmental issues, liver damage, immune system disruption, and an increased risk of certain cancers [2]. PFOA also harms the environment by affecting water ecosystems and land animals [5].

The U.S. Environmental Protection Agency (EPA) published a National Primary Drinking Water Regulation in April 2024 that sets individual maximum contaminant levels (MCLs) of 4.0 ng L^−1^ (ppt) for PFOA and for perfluorooctanesulfonic acid (PFOS). This limit was lowered from the 2016 lifetime health advisory of 70 ppt [6]. Following the EU Drinking Water Directive, Ireland applies a total PFAS limit of 0.5 µg L^−1^ and a sum of 20 PFAS of most concern of 0.1 µg L^−1^ [7].

PFOA is quantified by the reference methods liquid chromatography–tandem mass spectrometry (LC‐MS/MS) and gas chromatography–mass spectrometry (GC–MS) [8, 9, 10, 11]. These techniques provide excellent selectivity and reproducibility but can have practical limitations such as instrumentation cost, requisite laboratory infrastructure, labor‐intensive sample pretreatment, and typically poor suitability for routine in‐field use. Promising spectroscopic alternatives such as turn‐on or turn‐off fluorescence and surface‐enhanced Raman scattering (SERS) have also emerged for PFOA [12, 13, 14, 15, 16, 17] but can also be limited by selectivity, moderate reproducibility, and a need for sample pretreatment.

Electrochemical detection offers cost‐effectiveness, portability, and the possibility of on‐site analysis at trace levels. Pioneering technologies using molecularly imprinted polymer (MIP) electrodes have been successful approaches to selective electrochemical detection of PFAS, including poly(*o*‐phenylenediamine)‐templated MIPs yielding an 18 pM LOD for PFOS [18] and a 50 pM LOD for GenX [19]. Photoelectrochemical MIP hybrids have reached 0.1 nM (100 pM) for PFOA, and more recent architectures have lowered LODs for PFOA and PFOS into the subpicomolar regime [20, 21, 22].

At our lab, we devised an alternative approach to PFAS sensing based on zinc(II) terpyridine complexes (Zn–tpy) that can bind carboxylic acids by substitution of acetate ligands from their water‐soluble complexes of the type [Zn(tpy‐R)(OAc)_2_], where tpy‐R is a deriviative of 2,2′;6′,2″‐terpyridine [23]. Since PFOA also contains the carboxylate functional group, we aimed to explore its capability to react with Zn–tpy complexes toward electrochemical sensor development. Attractively, Zn–tpy complexes are well suited to electrochemical sensing due to their stability, reversible redox activity, and electron‐transfer capabilities [24].

Herein, we report the synthesis of two Zn–tpy complexes and characterize their reaction with PFOA by NMR and absorbance spectroscopies. This leads to an electrochemical PFOA sensor based on a Zn–tpy/Nafion‐modified GC electrode that achieves an LOD of 0.407 pM in phosphate‐buffered saline (PBS) and recovers 96%–98% of PFOA spiked into diluted Cork tap water. The Zn–PFOA capture and sense represents a simple approach to electrochemical screening of long‐chain perfluoroalkyl carboxylic acids.

## 2 Results and Discussion

### 2.1 Synthesis and Zn–PFOA Reaction Characterization

Two Zn–tepyridine complexes were synthesized and characterized following methods adapted from the literature [23, 25]. The complexes differ by bearing either diphenylamino‐ or diethylamino‐benzene substituents at the 4’ position of the terpyridine ligand to provide Zn–tpy–NPh_2_ and Zn–tpy–NEt_2,_ respectively (Figure 1). Reaction of the complexes with two equivalents of PFOA under stirring in methanol at room temperature led to the formation of a bright yellow precipitate in both cases that could be isolated by simple filtration for analysis. Complete substitution of the two acetate ligands by PFOA was evident from the ^1^H NMR spectra of the PFOA complexes by the disappearance of the acetate singlet at ca. 1.2 ppm, significant broading of the most downfield resonance at ca. 9.0 ppm, and other minor peak shifts of the terpyridine ligand signals (Figures 2a, S8, and S12). This was also confirmed by ^19^F NMR analysis that showed the expected perfluorooctyl peak pattern for the PFOA complexes (Figures S9 and S13). Interestingly, comparing the spectra of the Zn–PFOA complexes to published values for PFOA [26], a relative downfield shift was observed for the peak corresponding to the carboxylate‐adjacent CF_2_ position (ca. −117 ppm for Zn–PFOA vs. −119 ppm for PFOA), suggesting some releative deshielding at this position to support coordination of the carboxylate to zinc.

**FIGURE 1:**
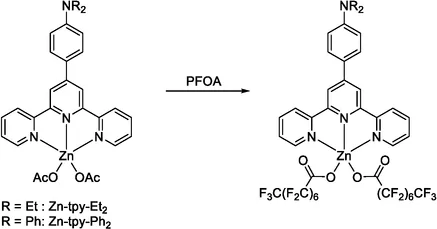
Chemical structures of the Zn–tpy complexes and their reaction with PFOA by substitution of the acetate ligands.

**FIGURE 2:**
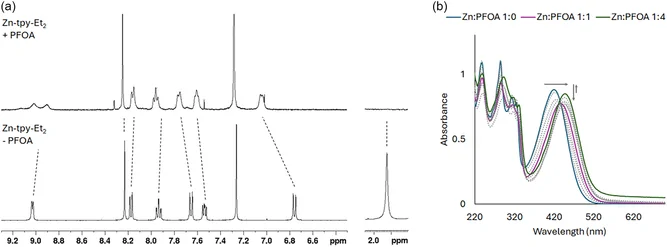
Spectroscopic changes upon reaction of PFOA with Zn–tpy complexes: (a) ^1^H NMR data and (b) UV/vis absorbance data.

The reaction of PFOA with Zn–tpy–NEt_2_ was further studied by absorbance spectroscopy using a series of solutions prepared in water with increasing concentration ratios of PFOA:Zn up to 4:1 (Figure 2b). Unfortunately, the diphenylamino complex, Zn–tpy–NPh_2_, exhibited poor solubility in water at the test concentration of 50 µM. In the absence of PFOA, the absorbance spectrum of Zn–tpy–NEt_2_ exhibits the expected peaks, with maxima at 238, 285, 314, and 325 nm due to intraligand electronic transitions of the terpyridine ligand, and a bathochromic band at 420 nm ascribed to a metal‐to‐ligand charge transfer (MLCT). Upon increasing the ratio of PFOA, there are clear spectral changes, with slight hypochromicity and redshifting for all bands. This is most apparent for the MLCT peak at 420 nm that gradually shifts to 447 nm as PFOA substitutes acetate ligands of the parent complex. This preliminary data suggests that such zinc terpyridine complexes could be developed as ratiometric optical sensors for PFOA, particularly if the spectroscopic peak separation can be increased. However, direct absorbance methods are limited by sensitivity, with Zn and PFOA concentrations in the micromolar range. Hence, attention turned to an electrochemical method that still uses the PFOA–Zn–terpyridine reaction, but now with the complex as a surface‐confined redox reporter. The poorly water‐soluble derivative Zn–tpy–NPh_2_ was selected specifically for this purpose because its low aqueous solubility favors surface confinement at GC.

### 2.2 Electrochemical Characterization of Modified Glassy Carbon Electrodes

Glassy carbon electrodes were modified with tpy–NPh_2_ ligand, Zn–tpy–NPh_2_ complex, Nafion alone, or Nafion over Zn–tpy–NPh_2_ and were characterized by CV using potassium ferrocyanide as the redox probe (10 mM [Fe(CN)_6_]^4−/3−^ in 0.1 M KCl). Moderate increases in peak current were observed for the ligand‐ and complex‐modified electrodes. Peak currents fell when a Nafion overlayer was present, consistent with partial Donnan exclusion of the anionic probe, although exclusion was less complete for Zn–tpy/Nafion than for Nafion alone (Figure 3).

**FIGURE 3:**
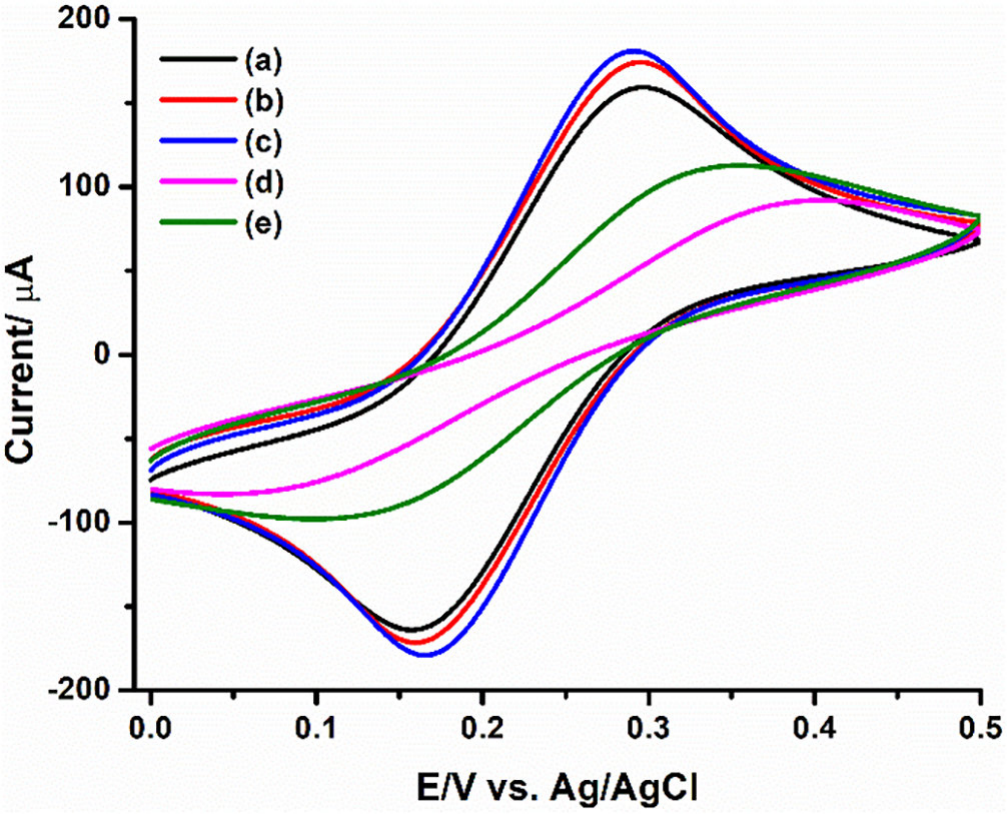
CVs of the different GC electrodes at a scan rate of 50 mV/s with different surface modifications were recorded in a solution of potassium ferrocyanide (10 mM [Fe(CN)_6_]^4−/3−^ in 0.1 M KCl) as the redox probe: (a) bare GC electrode, (b) tpy‐modified GC, (c) Zn–tpy‐modified GC, (d) Nafion‐modified GC, and (e) Zn–tpy/Nafion‐modified GC electrodes.

To rationalize the electrochemical behavior of the modified electrodes, it is important to clarify that Zn–tpy films were formed by cycling the cleaned GC electrode in aqueous suspension of 2 mM [Zn(tpy–NPh_2_)(OAc)_2_] containing 1 M KCl, from 0 to −1.2 V versus Ag/AgCl (10 cycles, 50 mV s^−1^). This potential range is well below the values needed for oxidative C─C coupling of a diphenylamino or triphenylamine group. That coupling starts only at about +0.8 V versus Ag/AgCl or higher after formation of an aminium radical cation [27, 28]. Indeed, when the same electrode is transferred into PBS, the ligand‐centered oxidation of the NPh_2_ group appears at about +1.4 V (Figure 5), a potential consistent with a monomeric diphenylamino unit and not the lower‐potential tetraphenylbenzidine couple that is seen after aryl–aryl coupling [27, 29]. We therefore assign film growth to potential‐assisted adsorption of the poorly water‐soluble complex on GC. Residual oxygen reduction and the start of hydrogen evolution also raise the local pH at the surface and can help the complex to accumulate. On this basis, the deposit is described as a thin, disordered film of [Zn(tpy–NPh_2_)(OAc)_2_] where acetate may be exchanged for water, chloride, or hydroxide ligands during cycling and rinsing. Nafion modification of the electrodes with a 5 µL drop of 0.025 wt% Nafion is expected to provide a thin, disordered, unannealed overlayer. Recast films prepared under these mild conditions are thin, poorly entangled, and often incomplete [30, 31, 32].

Electrochemical impedance spectroscopy (EIS) of the modified electrodes in 10 mM [Fe(CN)_6_]^4−^ ^/3−^/0.1 M KCl is shown in Figure 4 (fitted parameters are given in Table S1). Five traces were recorded: (a) bare GC, (b) tpy, (c) Zn–tpy, (d) Nafion, and (e) Zn–tpy/Nafion. On the scale of the figure, traces (b) and (c) overlap, so only four arcs are discernable. Qualitatively, bare GC, tpy, and Zn–tpy all show a small charge‐transfer resistance toward ferrocyanide. Nafion alone gives a large semicircle. This increase in *R*_ct_ is expected: the sulfonate groups of Nafion repel the anionic redox probe. The Zn–tpy/Nafion trace lies between the small arcs and the Nafion‐only semicircle, and the fitted *R*_ct_ falls from Nafion‐only to Zn–tpy/Nafion. Consistent with the residual Faradaic peak in Figure 3, this implies a heterogeneous Nafion overlayer atop the Zn–tpy coating that enables electrode access to the ferrocyanide probe.

**FIGURE 4:**
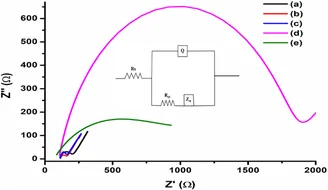
Nyquist plots of GC electrodes in 10 mM [Fe(CN)_6_]^4−^^/^^3−^ in 0.1 M KCl: (a) bare, (b) tpy, (c) Zn–tpy, (d) Nafion, and (e) Zn–tpy/Nafion. Traces (b) and (c) overlap on this scale. Inset: modified Randles circuit used for the fits in Table S1.

Interfacial capacitance is modeled as a constant‐phase element (CPE), not as an ideal capacitor. For bare, tpy, Zn–tpy, and Nafion‐only electrodes, the CPE exponent *n* is 0.72–0.81. For Zn–tpy/Nafion, *n* falls to 0.07, so the CPE parameter *Q* cannot be converted into a reliable double‐layer capacitance. The low exponent supports that this surface is heterogeneous.

### 2.3 Electrochemical Response to PFOA Solutions

The modified electrodes were exposed to 1 µM PFOA in 50 mM PBS (pH 7) at 50 mV s^−1^ (Figure 5). As expected, bare GC and Nafion‐only GC show no analytically useful peak. The tpy, Zn–tpy, and Zn–tpy/Nafion electrodes show an anodic peak at about +1.4 V versus Ag/AgCl, attributed to the ligand‐centered oxidation of the tpy diphenylamino group. Notably, the Nafion overlayer increases this current relative to Zn–tpy alone.

**FIGURE 5:**
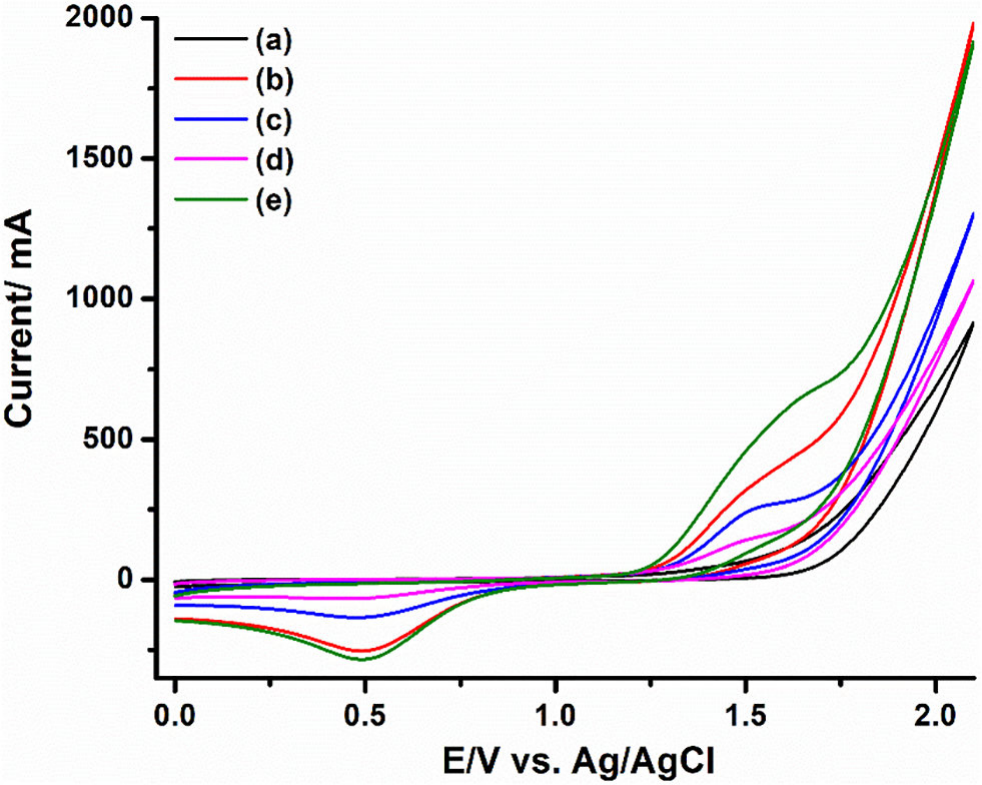
CVs of 1 µM PFOA at different GC modifications using 50 mM PBS, pH 7, and a scan rate of 50 mV/s: (a) bare, (b) tpy, (c) Zn–tpy, (d) Nafion, and (e) Zn–tpy/Nafion‐modified GC.

We propose that the thin and disordered Nafion overlayer has two roles. It anchors the poorly soluble Zn–tpy film on the electrode, and it can preconcentrate PFOA next to that film. Nafion is a phase‐separated ionomer where hydrated sulfonate domains coexist with a PTFE‐like fluorocarbon backbone [33, 34]. PFOA bears a long perfluoroalkyl chain that can interact with the fluorocarbon backbone of Nafion and this must overcome the expected Donnan exclusion effects of its anionic carboxylate head group in order to preconcentrate at the electrode and interact with the Zn–tpy film. The same fluorophilic uptake has been used to explain PFOA accumulation at perfluorinated coatings on carbon electrodes [34]. Interestingly, testing the sensor response with an octanoic‐acid control (Figure S3) supports this view; the hydrogenated analog gives almost no SWV response.

Calibration was performed under optimized conditions at the Zn–tpy/Nafion GC electrode. SWV parameters were amplitude 50 mV, frequency 10 Hz, and potential increment 10 mV, using the diagnostic anodic peak at approximately +1.4 V versus Ag/AgCl. Figure 6 shows representative SWV traces from 0.5 to 10 pM, together with the corresponding plot of peak current (*i*_p_) versus PFOA concentration. The linear regression equation was determined as *I* = 2.77 × 10^−6^ *C* + 5.4 × 10^−6^, *R* ^2^ = 0.995 (residual analysis in Table S2). A blank SWV of the Zn–tpy/Nafion electrode in PBS is shown with the 0.5 pM trace in Figure S2 so that the signal above background can be inspected. The LOD, calculated as 3*σ*/slope using the standard deviation of the blank‐regression intercept, is 0.407 pM; the limit of quantification (LOQ = 10*σ*/slope) is 1.36 pM. Intraelectrode repeatability (*n* = 3 successive SWV runs, same electrode) gave RSD = 0.94%. Interelectrode reproducibility (*n* = 3 independently prepared electrodes) gave RSD = 2.02%.

**FIGURE 6:**
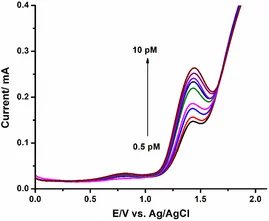
(a) SWV traces for 0.5–10 pM PFOA at Zn–tpy/Nafion GC in 50 mM PBS, pH 7. (b) Corresponding calibration plot of anodic peak current versus concentration with linear regression and *n* = 3 error bars. A blank trace is given in Figure S2.

Consistent with the above data, we propose that the analytical response to PFOA is via Nafion preconcentration at the electrode with subsequent carboxylate coordination to the Zn–tpy complex. The current increase with PFOA concentration (Figure 6) is likely to be in response to its accumulation at the Zn–tpy film, changing the local hydrophobic–fluorophilic environment to enhance the Faradaic current. Accordingly, the preliminary sensor data is promising as a class‐wide sensor for carboxylated PFAS since the proposed sensing mechanism is unlikely to discriminate related molecules like perfluorononanoic acid (PFNA). Going forward, to address mechanistic gaps, we intend to survey the substrate scope of the sensor and to pursue extensive characterization of the functional electrode surface pre‐ and postmeasurement to examine the factors that impact analytical performance (including overlayer continuity and Zn–tpy leaching studies).

### 2.4 Detection of PFOA in Drinking Water

Finally, we investigated the capability of Zn–tpy/Nafion GC electrodes to detect PFOA in drinking water. PFOA has been identified in Ireland around sites where aqueous film‐forming foams have been used, including surface water at the Dublin Fire Brigade Training Centre and Shannon Airport.

In the Cork region of Ireland, drinking water contains several ions that can appear in an SWV window of 0–1.8 V; Fe(II/III) is a typical example. A blank SWV of Cork tap water, diluted 1:100 into 50 mM PBS pH 7, was indistinguishable from the PBS background, indicating no detectable Faradaic interference from the ions present in that supply. Cork City drinking water is reported to be free from detectable PFOA and other PFAS. Therefore, the drinking water tested in our studies can be considered as a low‐organic, low‐PFAS tap‐water matrix.

Four test solutions were analyzed: (a) blank diluted tap water, and tap water spiked at (b) 5 pM, (c) 10 pM, and (d) 15 pM PFOA after dilution (Figure 7). Average recoveries were 96%–98% with RSD ≤ 3.5% (*n* = 3; Table 1). Note that the 15 pM spike lies above the 0.5–10 pM range of our calibration data (e.g., Figure 6) and is reported only as a recovery check, not as a calibrated unknown.

**FIGURE 7:**
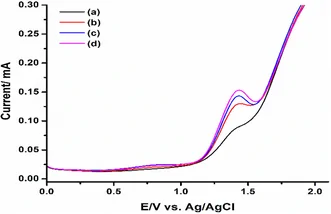
SWV of (a) 50 µL blank drinking water and PFOA spiked of (b) 5 pM, (c) 10 pM, and (d) 15 pM on the Zn–tpy/Nafion‐modified GC electrode, utilizing 50 mM PBS, pH 7.

**TABLE 1:** Analysis and recovery of PFOA in spiked drinking water.

Spiked PFOA, pM	Measured PFOA, pM	Recovery, %	RSD, %
5	4.8 ± 0.2	96	3.2
10	9.7 ± 0.3	97	3.1
15	14.7 ± 0.4	98	2.7

## 3 Conclusions

This study describes an electrochemical PFOA sensor at a GC electrode modified with a Zn–tpy complex film and a thin discontinuous Nafion overlayer that combine to enable preconcentration and a subsequent electrochemical response. Solution phase measurements by NMR and UV/vis spectroscopy support PFOA coordindation to the Zn–tpy complex, and this occurrence at the electrode is proposed to alter the local environment to elicit a current response. In PBS, the LOD was determined as 0.407 pM over a linear range of 0.5–10 pM, exhibiting competitive performance to reported MIP electrodes but with a narrower analytical range. Recoveries of 96%–98% were obtained for PFOA spiked into diluted Cork tap water. Combined, the preliminary data suggests Zn–tpy complexes in combination with selective coatings like Nafion may be applicable as class‐selective sensors for perfluoroalkyl carboxylic acids, exemplified herein using PFOA as the most common pollutant of the class. The next steps are to pursue dedicated characterization of the electrode surface, pre‐ and postexposure to PFOA and to examine PFAS substrate scope.

## 4 Experimental and Materials

### 4.1 General

All chemical reagents were sourced from Merck (Ireland) or Fluorochem (UK) and were used without further purification. NMR analysis was carried out on a Bruker Avance 400 MHz spectrometer at room temperature using deuterated solvents, calibrated against residual solvent signals. Absorbance spectroscopy was performed using a Cary 50 Scan UV‍–‍visible spectrophotometer.

### 4.2 Synthesis

Synthetic procedures for the ligands and complexes are adapted from published protocols, and NMR data provided herein conforms to the literature [25, 35].

#### 4.2.1 tpy–NEt_2_ Ligand

4‐(Diethylamino)benzaldehyde (1760 mg, 9.93 mmol), 2‐acetylpyridine (2.8 mL, 24.96 mmol), potassium hydroxide (1970 g, 35.11 mmol), ammonium hydroxide (28% aq., 30 mL), and ethanol (40 mL) were heated at reflux for 4 h. After cooling, the precipitate that formed was isolated by filtration and washed with ethanol (2 × 10 mL) to yield a crude solid. Recrystallization from ethanol (15 mL) provided the pure ligand. Yield: yellow solid, 1977 mg (56%). ^1^H NMR (400 MHz, CDCl_3_) *δ* (ppm): 8.64–8.78 (m, 6H), 7.86 (m, 4H), 7.33 (dd, 2H), 6.78 (d, 2 H), 3.43 (q, 4H), 1.22 (t, 6H).

#### 4.2.2 tpy–NPh_2_ Ligand

2‐Acetylpyridine (710 mg, 5.87 mmol) and 4‐(diphenylamino)benzaldehyde (400 mg, 1.46 mmol) were stirred in 30 mL of ethanol for 5 min. Potassium hydroxide (328 mg) was then added, followed by ammonium hydroxide (28% aq., 10 mL), and stirring was continued for 3 days. The orange‐yellow suspension that formed was filtered and washed with ethanol to provide crude product that was twice recrystalized from ethanol to afford pure product. Yield: yellow‐orange solid, 280 mg (40%). ^1^H NMR (400 MHz, CDCl_3_) *δ* (ppm): 8.66–8.80 (m, 6H), 7.91 (t, 2H), 7.82 (d, 2H), 7.37 (t, 2H), 7.27–7.33 (m, 4H), 7.13–7.22 (m, 6H), 7.08 (t, 2 H).

#### 4.2.3 [Zn(tpy–NEt_2_)(OAc)_2_]

tpy–NEt_2_ (500 mg, 1.31 mmol) and Zn(OAc)_2_ (241 mg, 1.31 mmol) were stirred in methanol at room temperature for 24 h, and then the solvent was removed by evaporation under reduced pressure. The crude residue was purified by recrystalization from acetone. Yield: yellow solid, 667 mg (90%). Yield: yellow solid. ^1^H NMR (400 MHz, CDCl_3_) *δ* (ppm): 9.03 (d, 2H), 8.16–8.24 (m, 4H), 7.93 (t, 2H), 7.65 (d, 2H), 7.54 (dd, 2H), 6.75 (d, 2H), 3.45 (q, 4H), 1.92 (s, 6H), 1.25 (t, 6H).

#### 4.2.4 [Zn(tpy–NPh_2_)(OAc)_2_]

tpy–NPh_2_ (200 mg, 0.42 mmol) and Zn(OAc)_2_ (77 mg, 0.42 mmol) were stirred in 10 mL methanol at room temperature overnight. The yellow suspension that formed was filtered, washed with acetone, and dried briefly in an oven. Yield: orange solid, 197 mg (71%). ^1^H NMR (400 MHz, CDCl_3_) *δ* (ppm): 9.04 (d, 2H), 8.28 (m, 2H), 8.21 (d, 2H), 8.00 (q, 2H), 7.56–7.64 (m, 4H), 7.35 (t, 4H), 7.12–7.21 (m, 8H), 1.92 (s, 6H).

#### 4.2.5 [Zn(tpy–NEt_2_)(PFOA)_2_]

[Zn(tpy–NEt_2_)(OAc)_2_] (100 mg, 0.18 mmol), PFOA (147 mg, 0.35 mmol), and methanol (10 mL) were stirred at room temperature for 2 h. The resulting precipitate was isolated by filtration and washed with methanol to yield a bright yellow solid. ^1^H NMR (400 MHz, CDCl_3_) *δ* (ppm): 8.93 (d, 2H), 8.20 (s, 2H), 8.10 (d, 2H), 7.90 (t, 2H), 7.70 (s, 2H), 7.57 (s, 2H), 6.96 (s, 2H), 3.48 (q, 4H), 1.23 (t, 6H). ^19^F NMR (CDCl_3_) *δ* (ppm): −80.80, −117.42, −121.68, −121.07, −122.71, −126.12.

#### 4.2.6 [Zn(tpy–NPh_2_)(PFOA)_2_]

Synthesis was performed as for [Zn(tpy–NEt_2_)(PFOA)_2_] using [Zn(tpy–NPh_2_)(OAc)_2_] to yield a yellow solid. ^1^H NMR (400 MHz, CDCl_3_) *δ* (ppm): 8.80–9.20 (broad t, 2H), 8.32 (s, 2H), 8.25 (d, 2H), 8.08 (t, 2H), 7.65 (t, 4H), 7.36 (m, 4 H), 7.13–7.23 (m, 8H). ^19^F NMR (CDCl_3_) *δ* (ppm): −80.81, −117.46, −121.72, −122.11, −122.78, −126.14.

### 4.3 Electrochemical Apparatus and Measurements

EIS was performed using a CHI660E electrochemical workstation (CH Instrument, Austin, TX), with an AC frequency range from 1 Hz to 100 kHz at +0.2 V. Amperometric measurements (*i−t* curves), CV, and SWV were carried out using a CHI1040A electrochemical workstation (CH Instrument, Austin, TX). All electrochemical measurements took place at room temperature. The electrochemical cell consisted of a GC working electrode (Windsor Scientific, Slough Berkshire, UK), a Pt wire counter electrode (Sigma–Aldrich, Dublin, Ireland), and a silver chloride (Ag/AgCl/3 M KCl) reference electrode (BASi Analytical Instruments, West Lafayette, IN).

### 4.4 Fabrication of the Zn–tpy/Nafion‐Modified Glassy Carbon Electrode

The GC electrode was polished using wet Nylon and MasterTex papers, sequentially treated with 0.3 µm and then 0.05 µm alumina slurries, and followed by sonication in ethanol or propanol (5 min), and then deionized water (10 min). A 2 mM solution of Zn–tpy–NPh_2_ was prepared in 1 M aqueous KCl with gentle heating and stirring. The cleaned electrode was immersed in this solution and cycled from 0 to −1.2 V versus Ag/AgCl at 50 mV s^−^^1^ for 10 cycles (20 segments). This step deposits a thin film of the complex by potential‐assisted adsorption. The electrode was rinsed with deionized water and dried with nitrogen. Nafion perfluorinated resin solution (5 wt% in a mixture of lower aliphatic alcohols and water; acid form; Merck) was diluted with ethanol to 0.025 wt% immediately before drop‐casting. Then, 5 µL was applied to the Zn–tpy‐modified GC electrode and allowed to dry for 30 min in a fume hood at ambient temperature, without thermal annealing.

### 4.5 Samples of Drinking Water

Drinking water samples were collected from the tap supply in the laboratory at University College Cork, Ireland, and used without filtration. For method validation, 50 µL of tap water was added to 5 mL of 50 mM PBS pH 7 (1:100 dilution) in the cell, after which PFOA was spiked. The dilution reduces matrix effects while keeping the spike concentrations inside or near the calibration range.

## Funding

This study was supported by the Royal Society of Chemistry (E22‐2342194673) and Deanship of Scientific Research, King Faisal University (KFU254750).

## Conflicts of Interest

The authors declare no conflicts of interest.

## Supporting information

Supplementary Material

## Data Availability

The data that support the findings of this study are available in the Supporting Information of this article.
