# Supplementary material for: Electrochemical Detection of Perfluorooctanoic Acid by a Zn–Terpyridine and Nafion‐Modified Glassy Carbon Electrode
Source: Chempluschem. 2026 Oct 20;91(10):e70272. doi: 10.1002/cplu.70272 (PMC13683051; doi:10.1002/cplu.70272)
Supplement: Supplementary file 1 [file CPLU-91-e70272-s001.pdf]

Supporting Information

Electrochemical Detection of PFOA by a Zn–Terpyridine and Nafion Modified Glassy Carbon Electrode

Alyah Buzid [1] , Kim J. Keohane [2] , Sarah Cramp [2] , and Christopher S. Burke* [2] .

[1]Department of Chemistry, College of Science, King Faisal University, PO Box 380, Al-Ahsa 31982, Saudi Arabia.

[2]School of Chemistry and Analytical & Biological Chemistry Research Facility (ABCRF), University College Cork, College Road, T12 YN60 Cork, Ireland.

***Corresponding Author:**

Christopher S. Burke, christopherburke@ucc.ie

1. Electrochemical Data

Table S1. Fitted EIS parameters from the modified Randles circuit.
Units: Rs and Rct in Ω; CPE Q in S·s^n (or Ω−1 s^n); n dimensionless; Warburg in Ω s−1/2; χ^2 dimensionless. Values are comparative fit outputs for one representative electrode of each type, not physical constants of the bulk electrolyte.

**Electrode**	**Rs / Ω**	**CPE Q / S s^n**	**n**	**Rct / Ω**	**W / Ω s−1/2**	**χ^2**
**Bare**	109.3	1.284E-5	0.7209	91.77	0.002417	0.0003406
**Ligand**	116.8	6.861E-6	0.8148	59.66	0.00282	0.0001366
**Zn Complex**	112.1	1.623E-5	0.735	46.58	0.002761	0.0001734
**Nafion**	103.4	1.284E-6	0.8142	1737	0.001362	0.0007758
**Nafion-Zn**	57.31	0.0009194	0.0712	0.8535	4.124E-5	0.002575

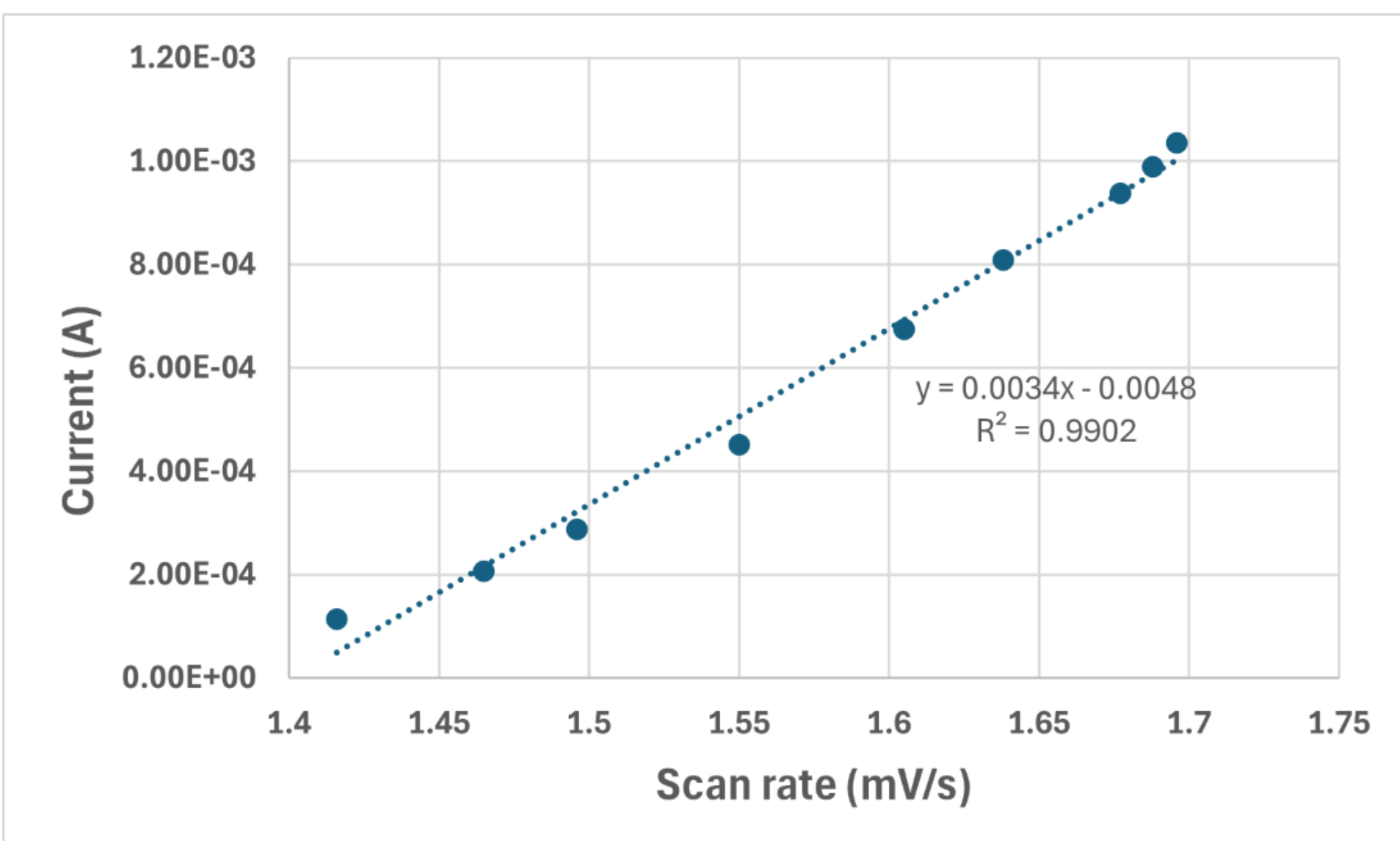

Figure S1. Scan rate (10–1000 mV s^{-1}) versus current for PFOA at Zn-tpy/Nafion GC in 50 mM PBS, pH 7. This plot is a single-run trend; independent replicates and error bars were not recorded.

Table S2. PFOA calibration on Zn-tpy/Nafion GC by SWV (0.5–10 pM).
LOD = 3σ_intercept / slope = 0.407 pM; LOQ = 10σ_intercept / slope = 1.36 pM. Repeatability RSD = 0.94% (n = 3 successive runs, same electrode); reproducibility RSD = 2.02% (n = 3 independently prepared electrodes).

Range (pM)	0.5-10
Correlation coefficient R^2	0.995
Linear equation (I = current, C= concentration (pM))	$I = 2.77 \times 10^{-6} C + 5.4 \times 10^{-6}$
The standard error for intercept	3.76×10^{-7}
The standard error for slope	6.34×10^{-8}
Calculated limit of detection (pM)	0.407
Calculated limit of quantification (pM)	1.36
Repeatability (relative standard deviation, RSD %)	0.94
Reproducibility (RSD %)	2.02

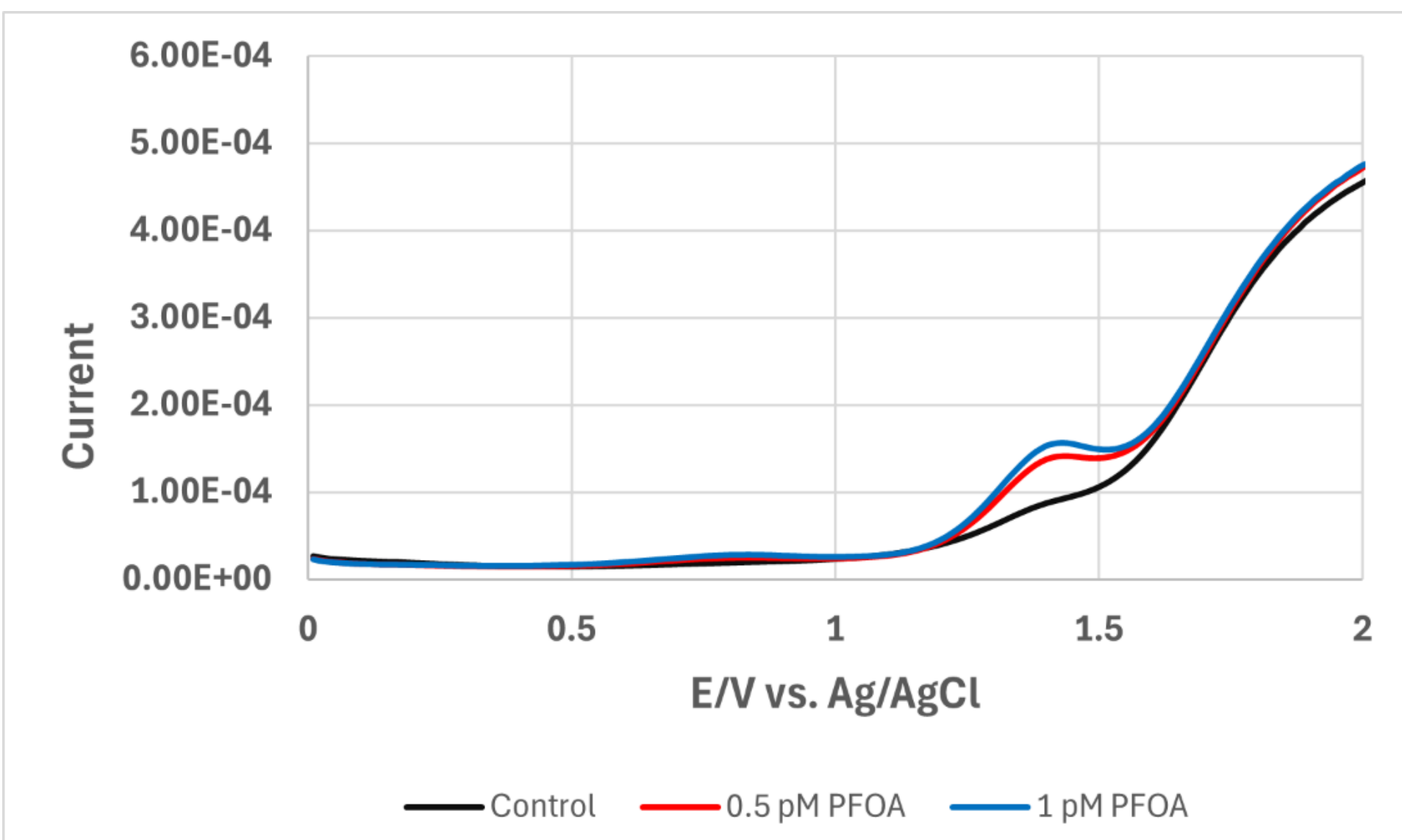

Figure S2. SWV of the PBS blank (black), 0.5 pM PFOA (red) and 1 pM PFOA (blue) at Zn-tpy/Nafion GC in 50 mM PBS, pH 7. The blank is the background current against which the 0.5 pM signal is judged.

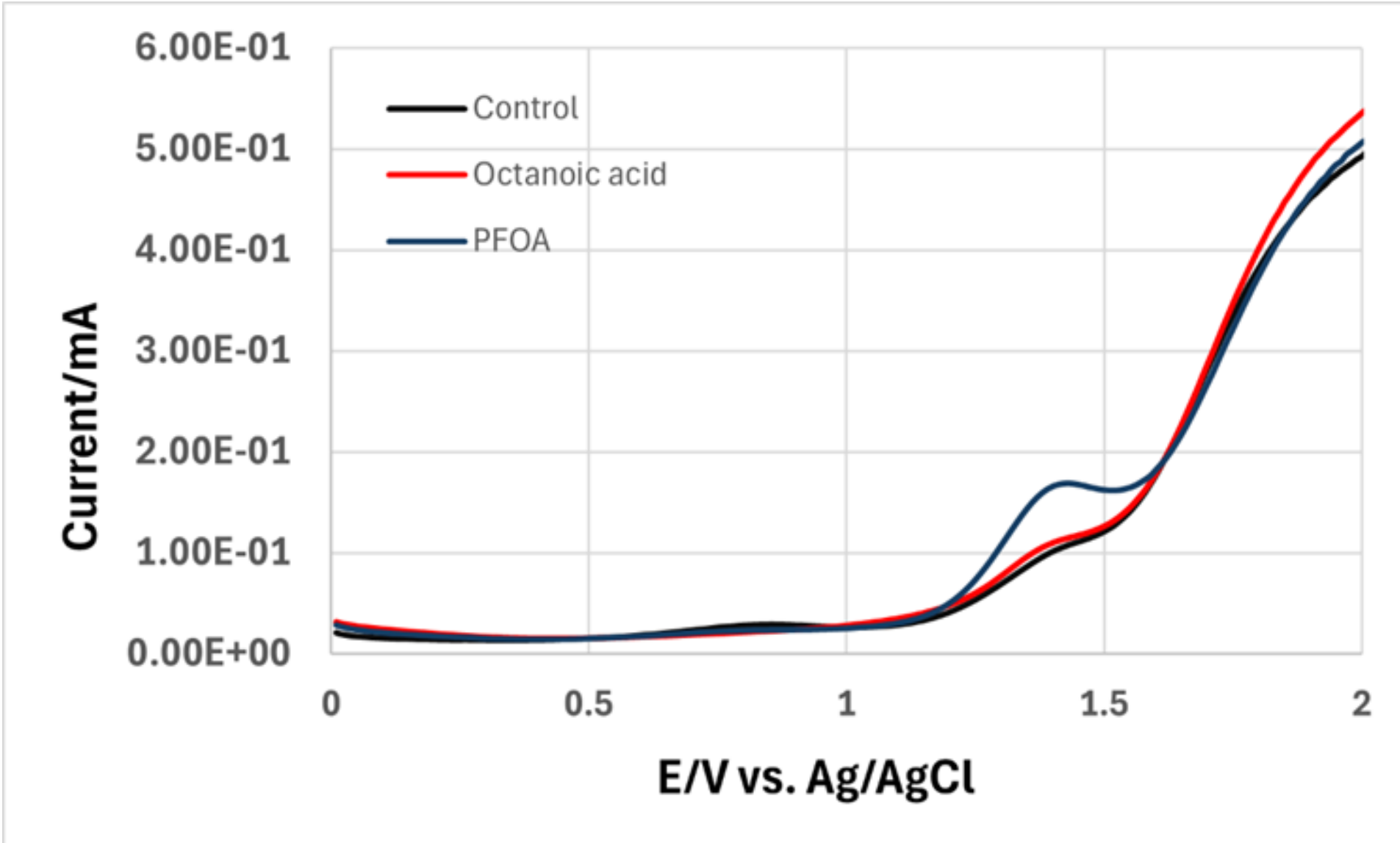

Figure S3. SWV of the control (black line), 1 mM octanoic acid (red line), and 50 pM perfluorooctanoic acid (PFOA, blue line) recorded on a Zn-tpy/Nafion-modified glassy carbon (GC) electrode. Measurements were performed in 50 mM phosphate-buffered saline (PBS) at pH 7.

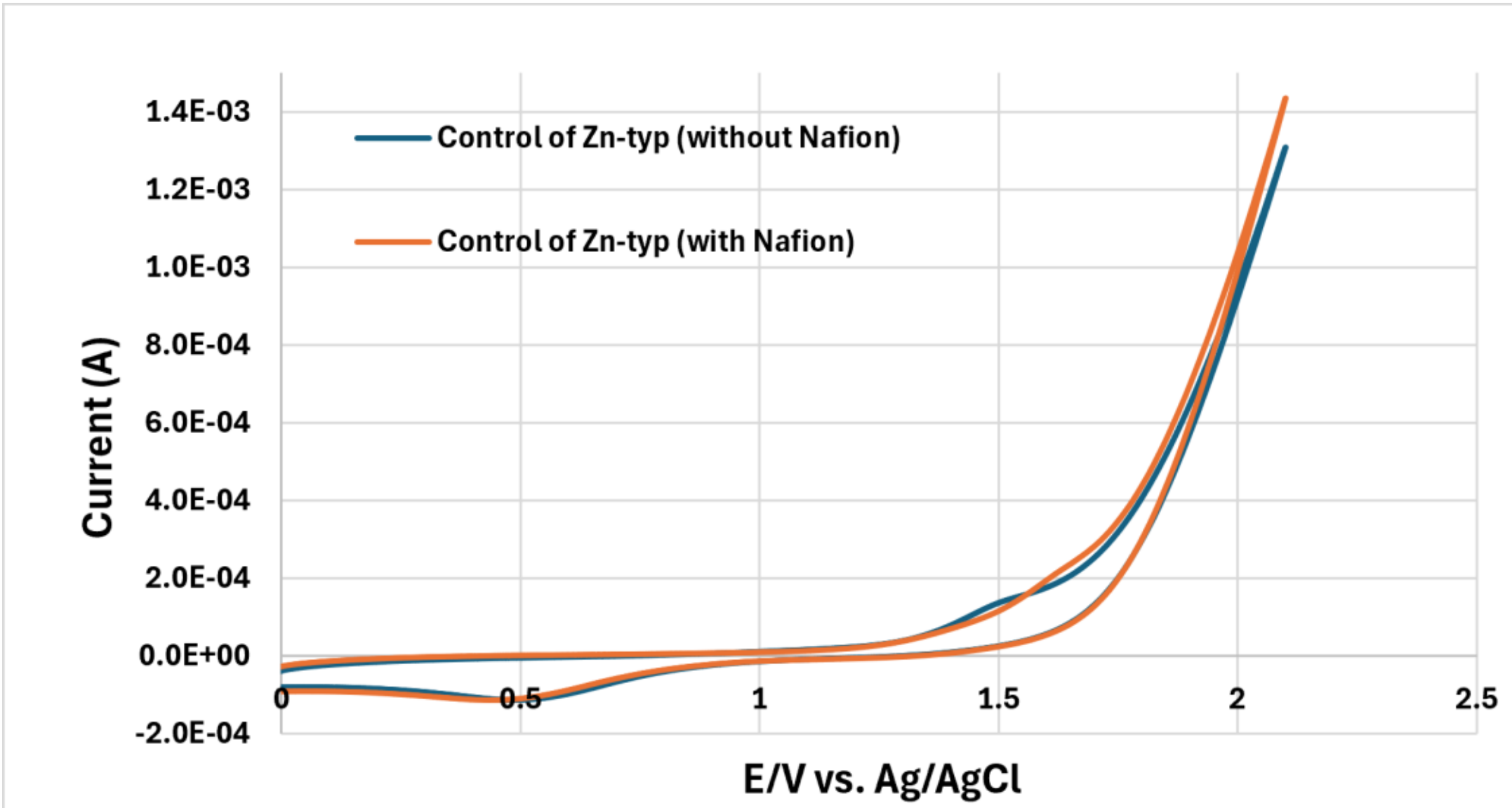

Figure S4 – CVs of Zn-tpy-Ph_2 complex modified glassy carbon electrode, with and without Nafion overlayer.

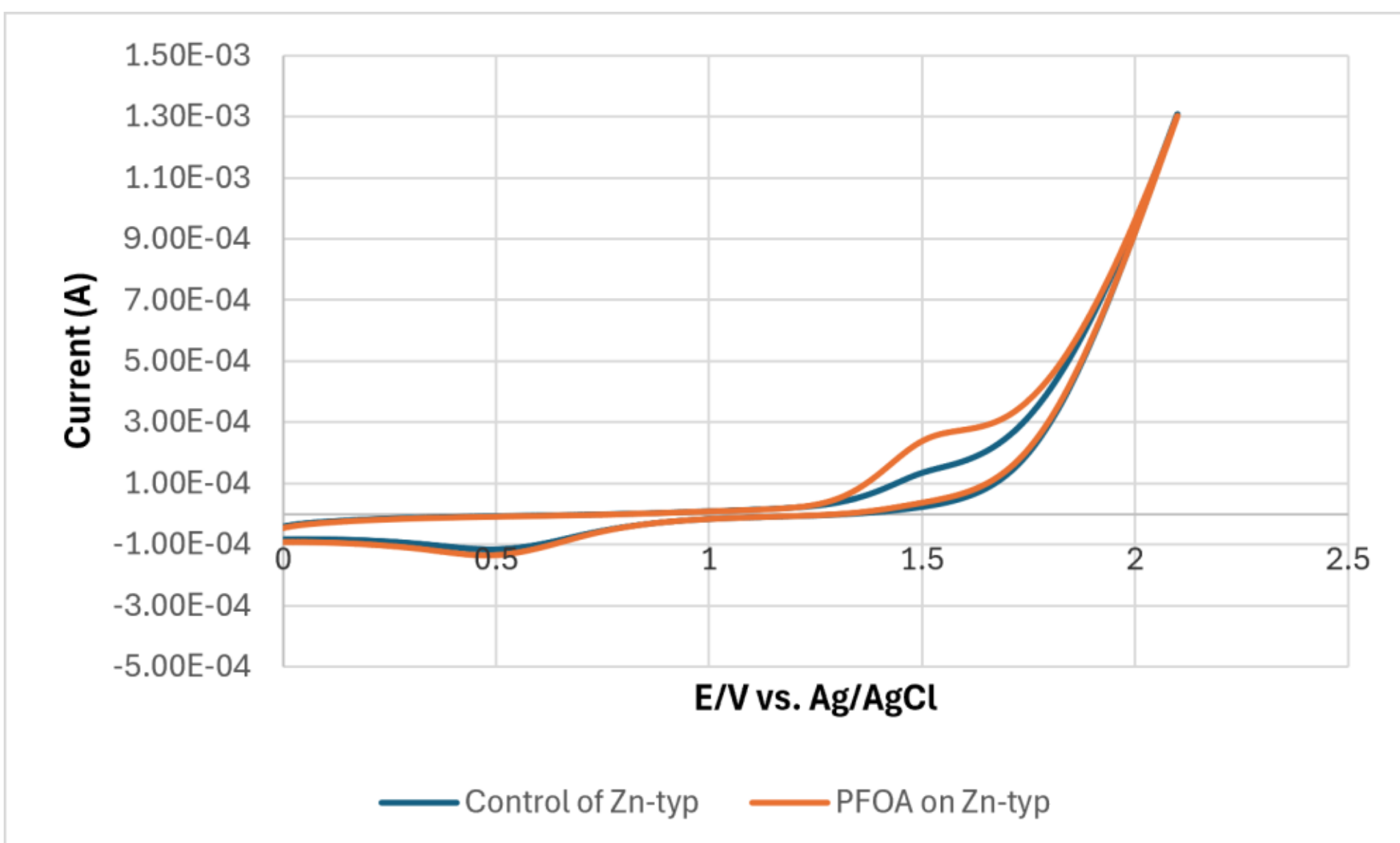

Figure S5 – CVs of Zn-tpy-Ph_2 complex modified glassy carbon electrode, in the presence and absence of PFOA (1 µM).

2. NMR Spectra

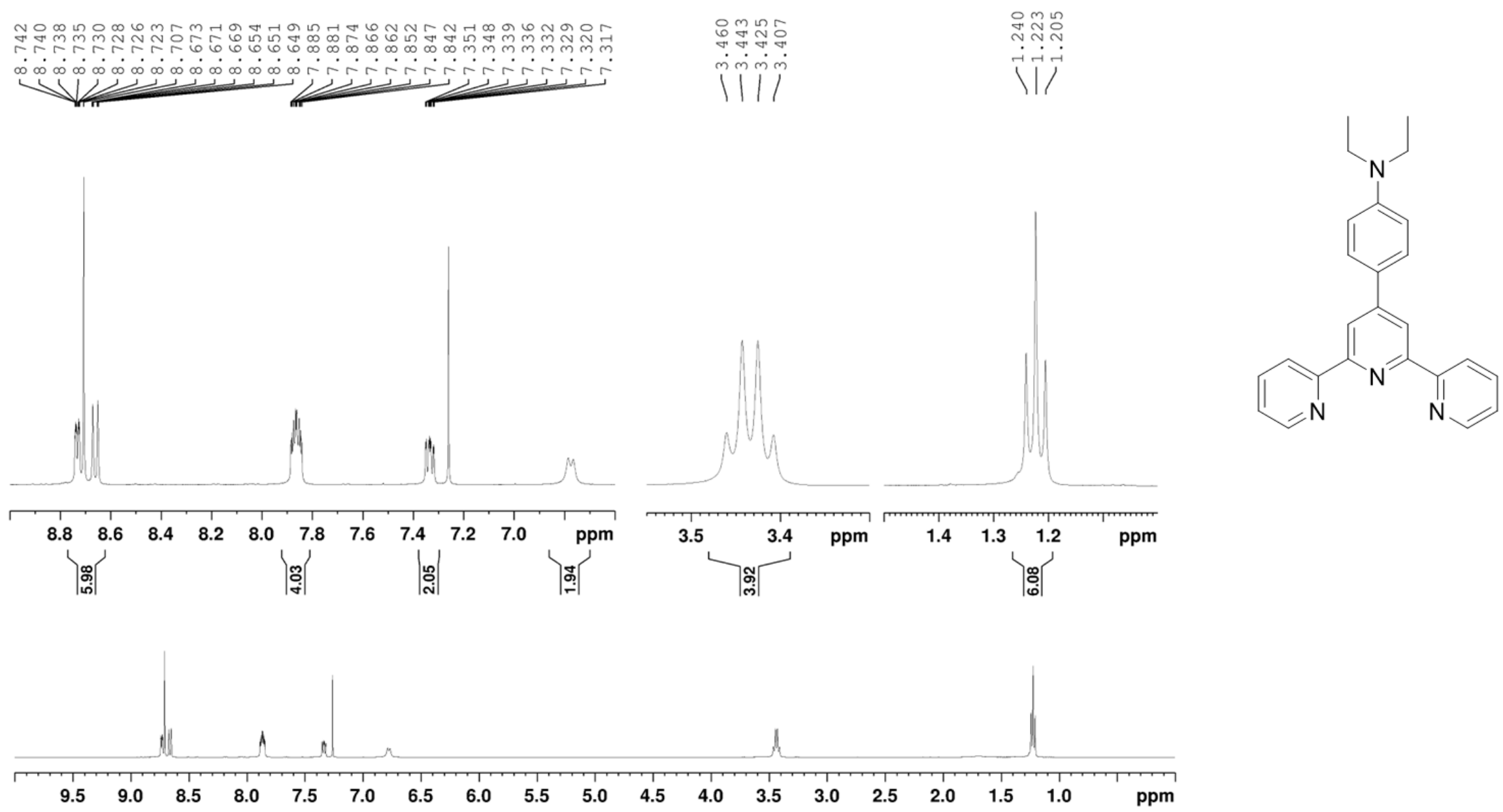

Figure S6. ^{1}H NMR (400 MHz, $CDCl_3$) spectrum of tpy-NEt_2.

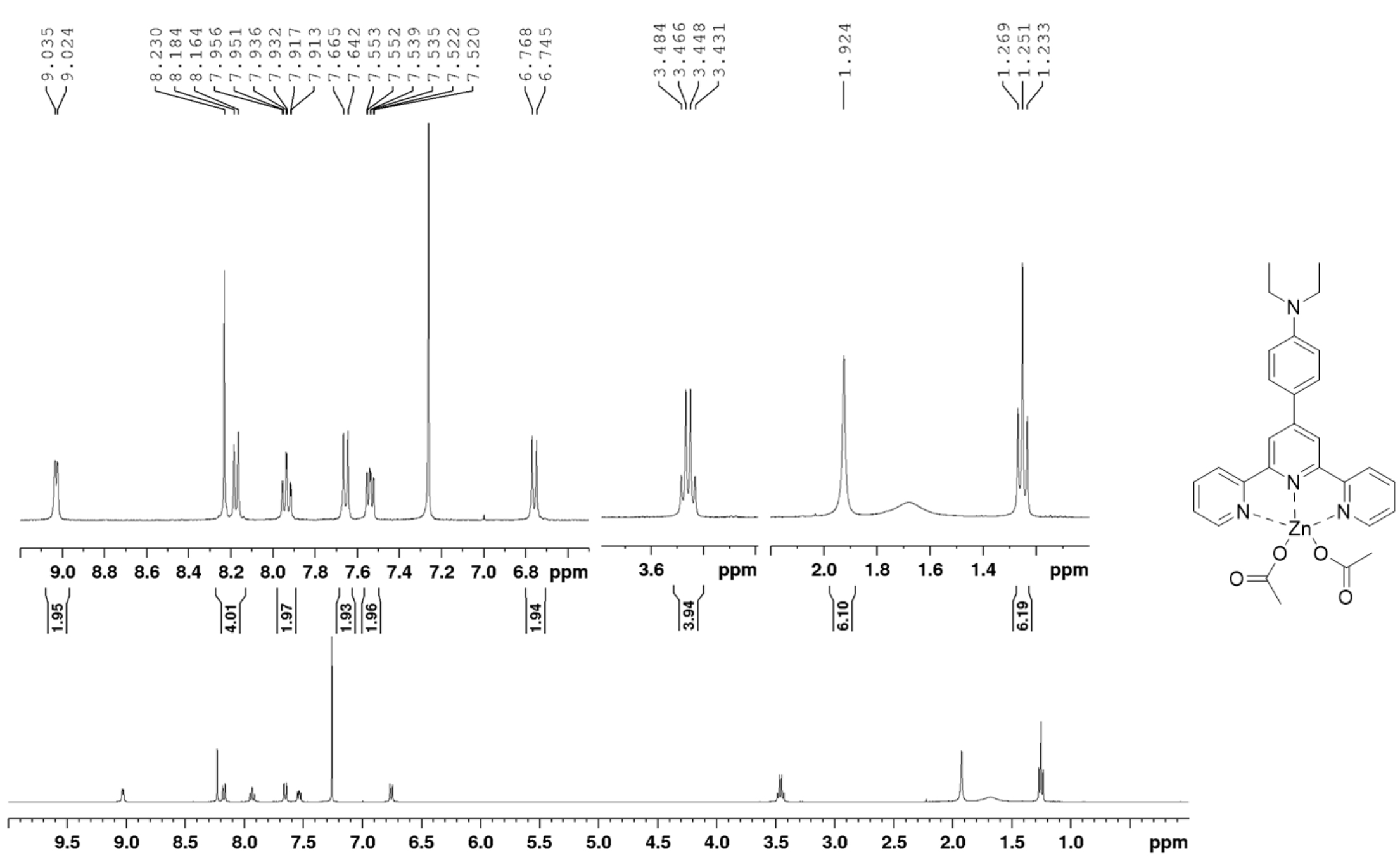

Figure S7. ^{1}H NMR (400 MHz, $CDCl_3$) spectrum of [Zn(tpy-NEt_2)$(OAc)_2$].

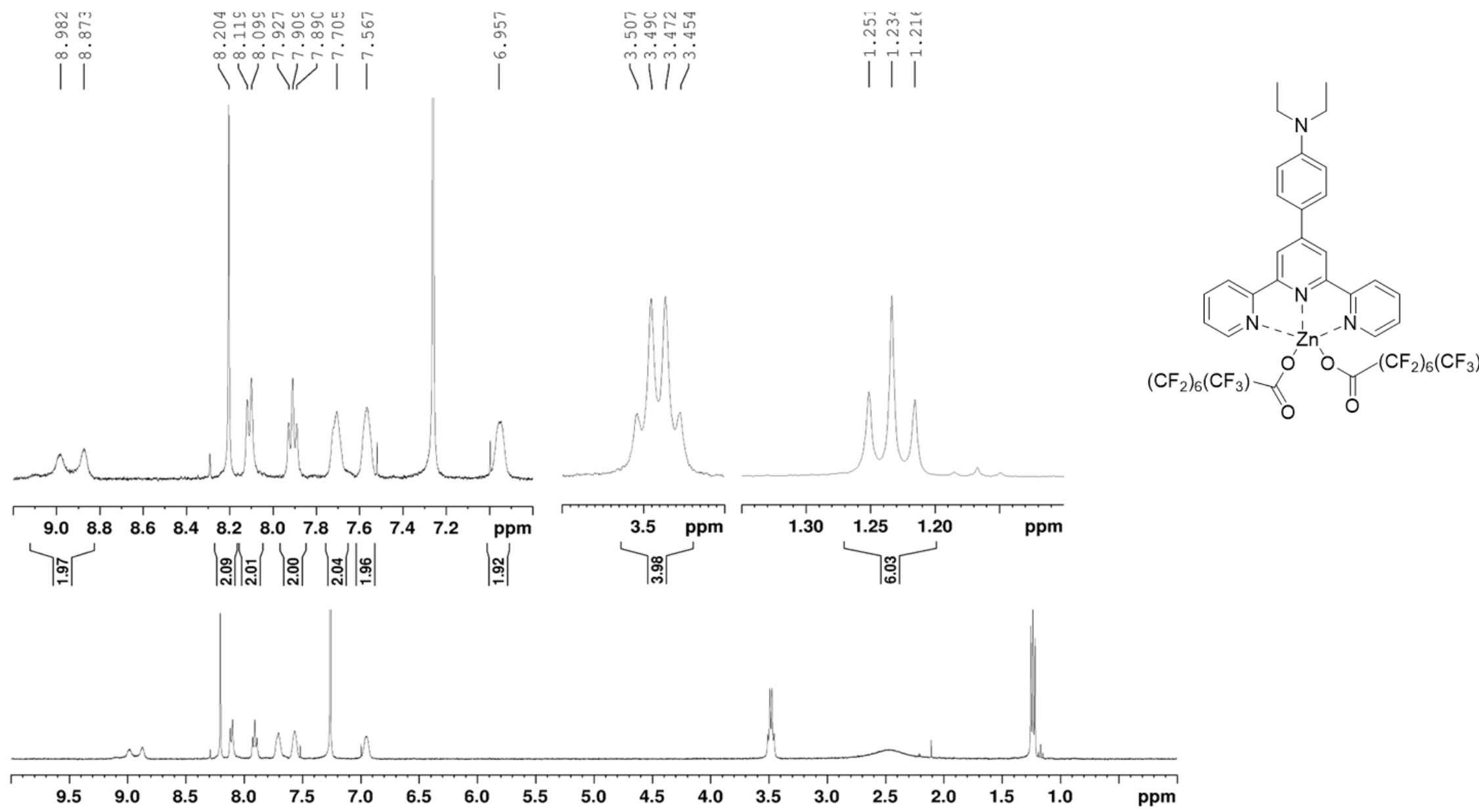

Figure S8. ^{1}H NMR (400 MHz, $CDCl_3$) spectrum of [Zn(tpy-NEt_2)$(PFOA)_2$].

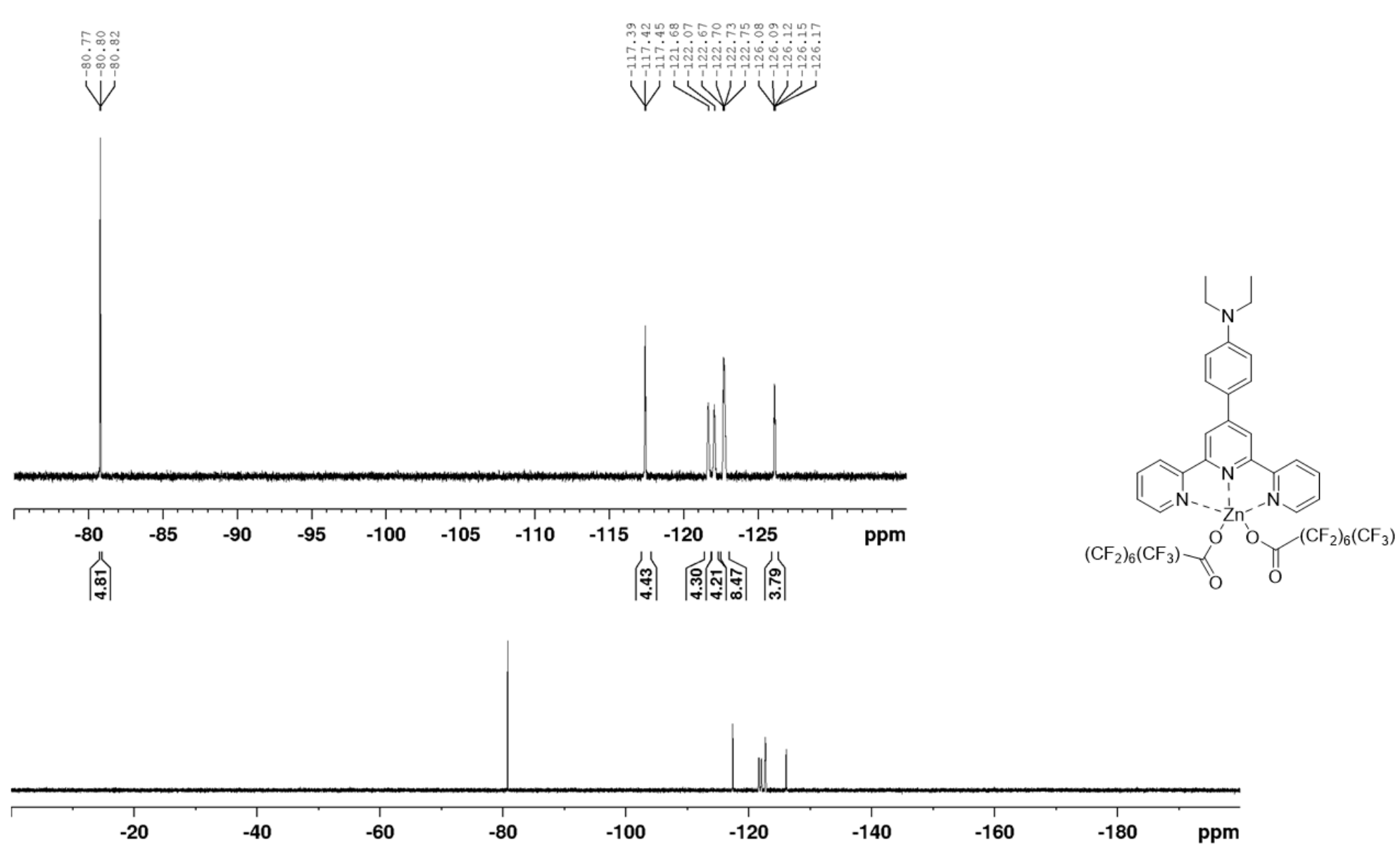

Figure S9. ^{19}F NMR (400 MHz, $CDCl_3$) spectrum of [Zn(tpy-NEt_2)$(PFOA)_2$].

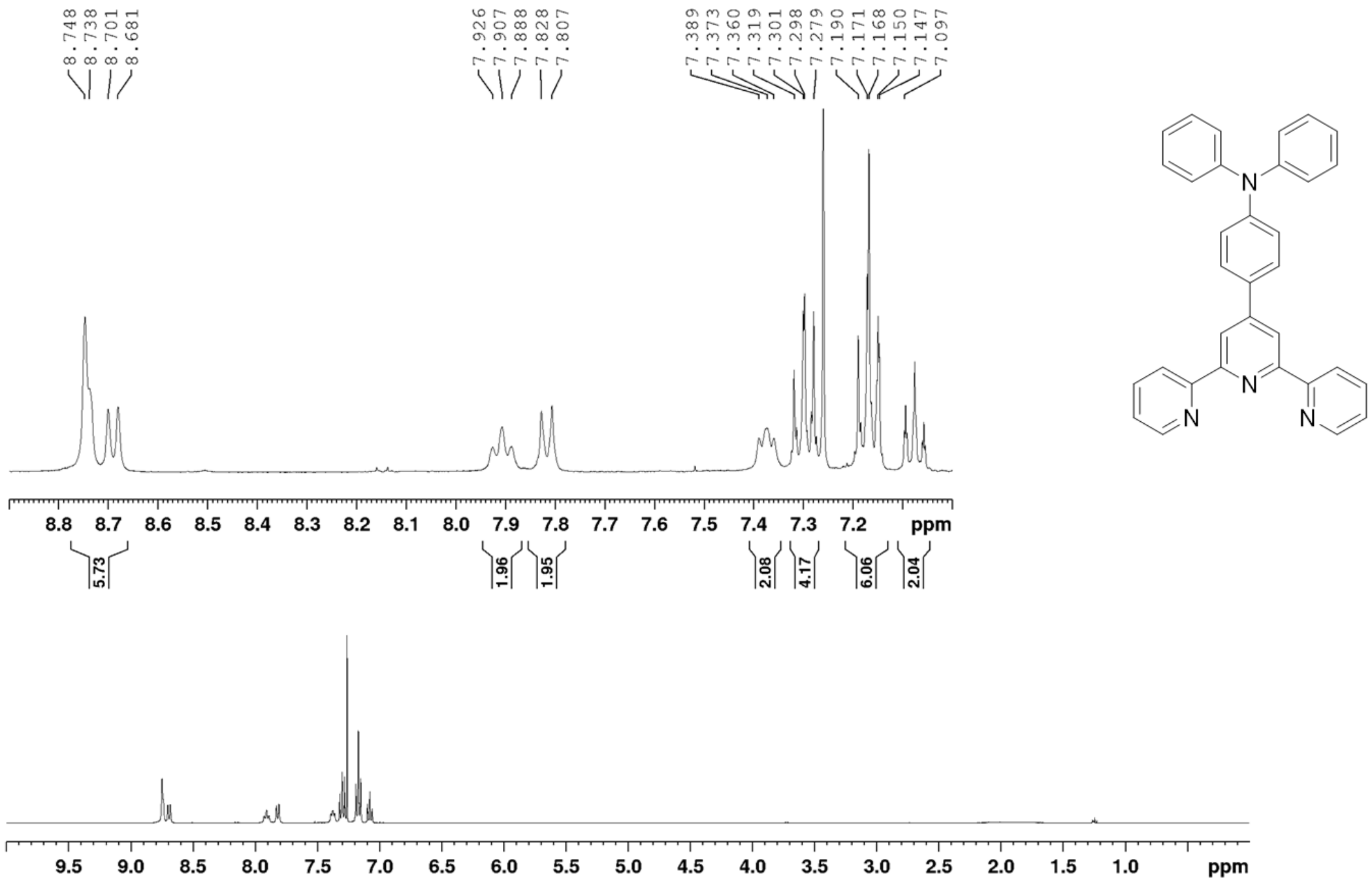

Figure S10. ^{1}H NMR (400 MHz, $CDCl_3$) spectrum of tpy-NPh_2.

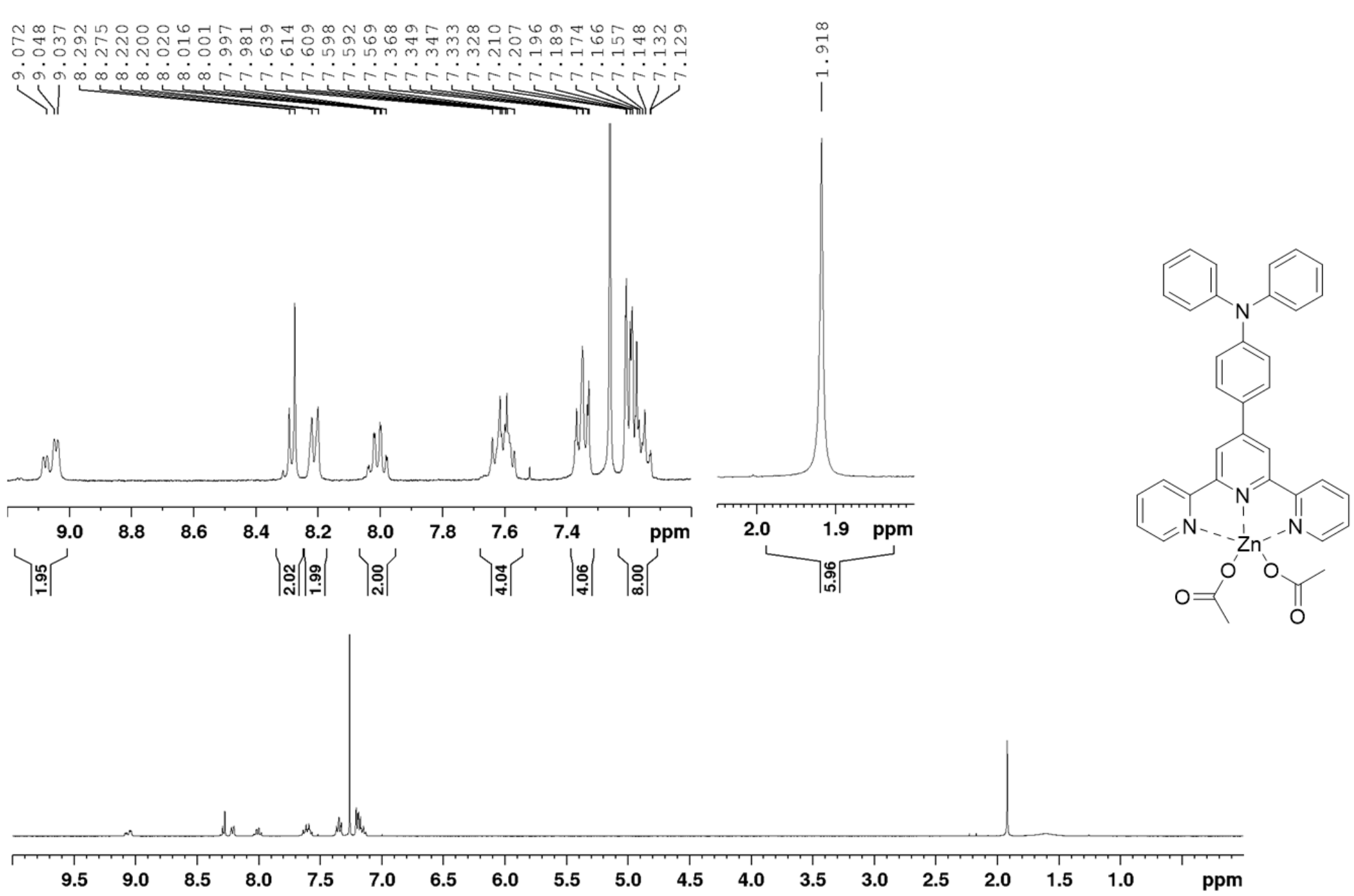

Figure S11. ^{1}H NMR (400 MHz, $CDCl_3$) spectrum of [Zn(tpy-NPh_2)$(OAc)_2$].

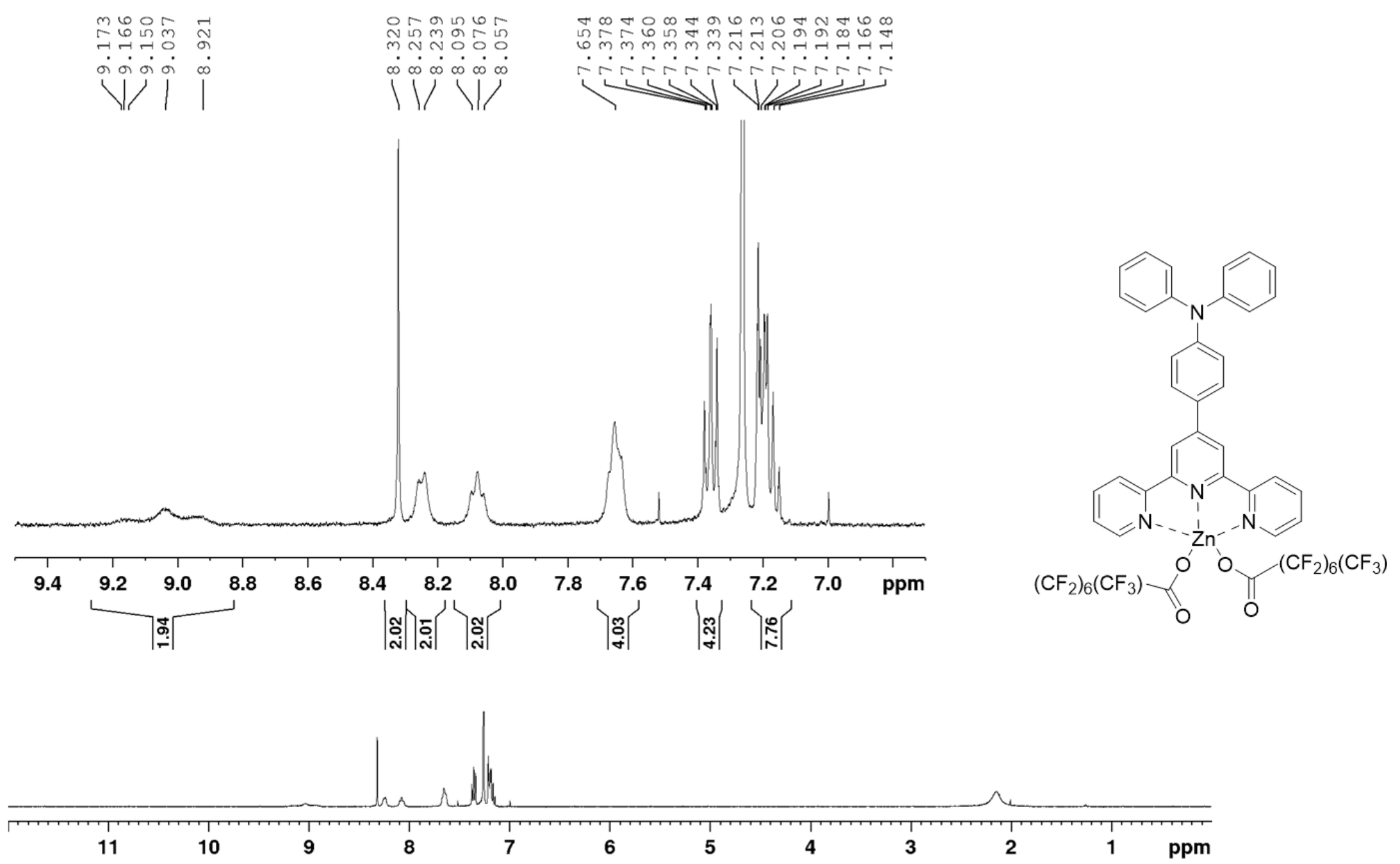

Figure S12. ^{1}H NMR (400 MHz, $CDCl_3$) spectrum of $[Zn(tpy\text{-}NPh_2)(PFOA)_2]$.

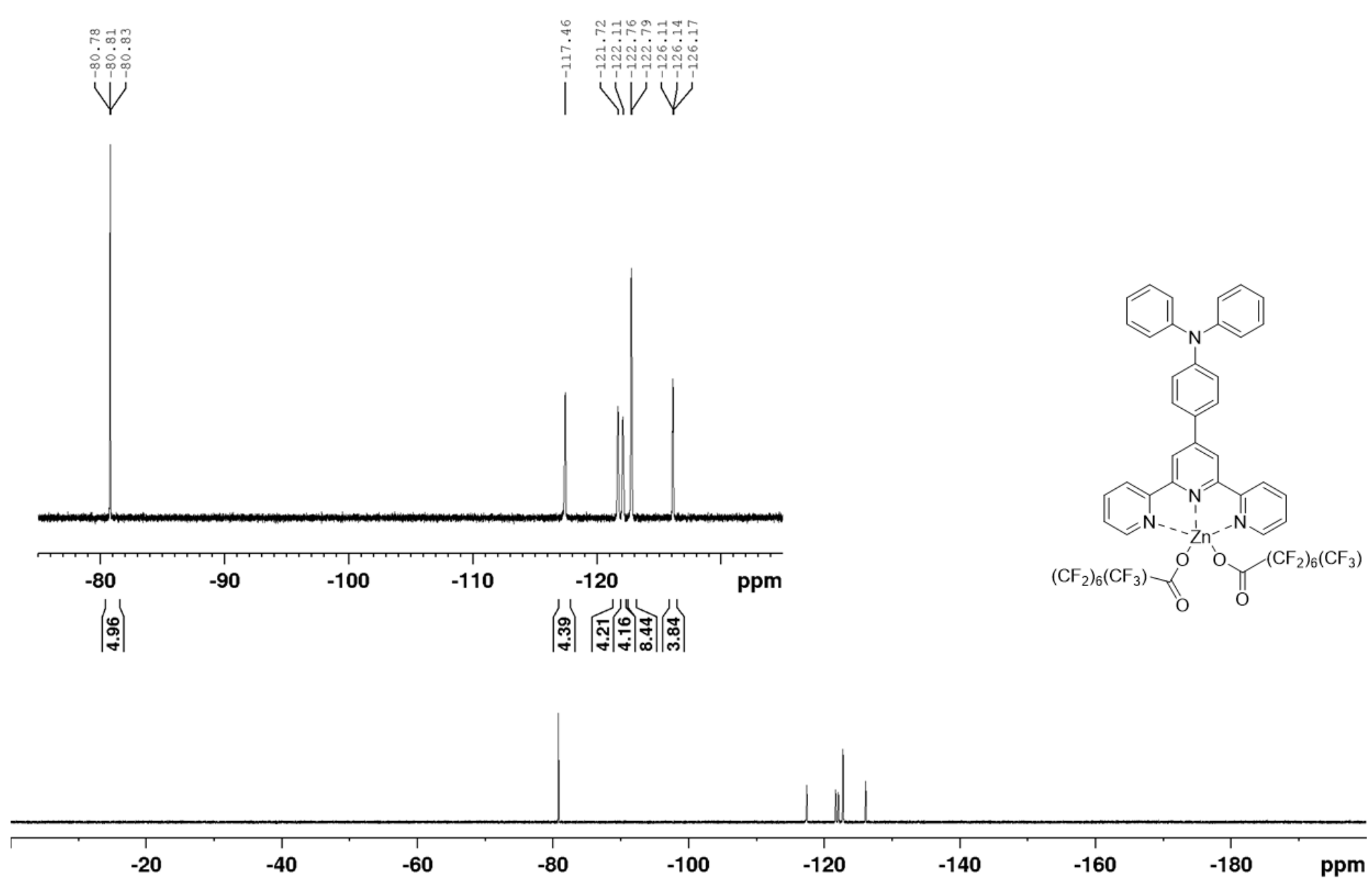

Figure S13. ^{19}F NMR (400 MHz, $CDCl_3$) spectrum of $[Zn(tpy\text{-}NPh_2)(PFOA)_2]$.

3. Absorbance Spectra

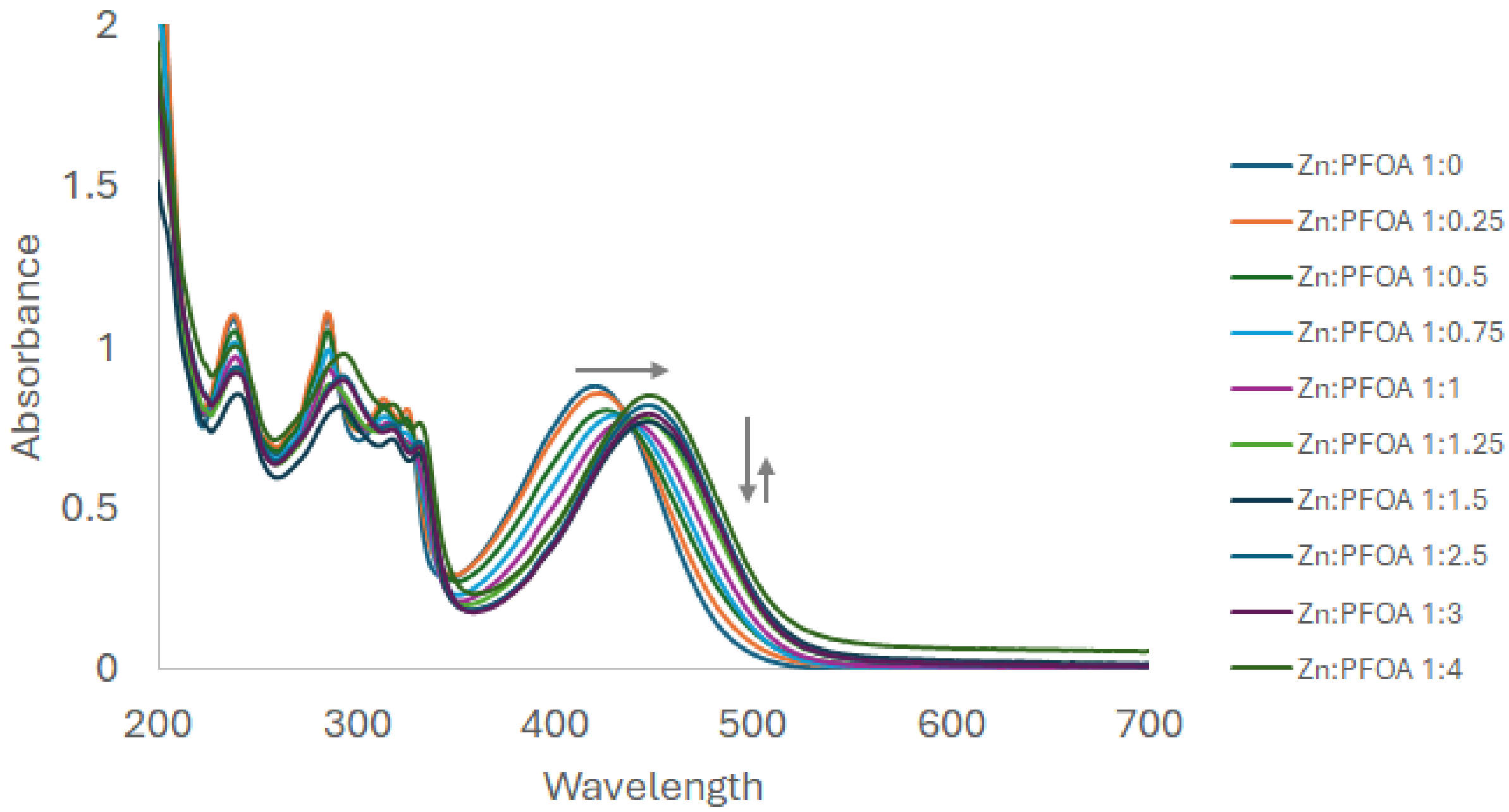

Figure S14. UV/Vis absorbance spectra of [Zn(tpy-NEt_2)$(OAc)_2$] in water (50 μM) with increasing relative concentration of PFOA (0–4 molar equivalents.
